# Usefulness of ^68^Ga-DOTATOC PET/CT to localize the culprit tumor inducing osteomalacia

**DOI:** 10.1038/s41598-021-81491-2

**Published:** 2021-01-19

**Authors:** Dong Yun Lee, Seung Hun Lee, Beom-Jun Kim, Wanlim Kim, Pil Whan Yoon, Sang Ju Lee, Seung Jun Oh, Jung-Min Koh, Jin-Sook Ryu

**Affiliations:** 1 Department of Nuclear Medicine, Asan Medical Center, University of Ulsan College of Medicine, Seoul, South Korea; 2 Division of Endocrinology and Metabolism, Asan Medical Center, University of Ulsan College of Medicine, Seoul, South Korea; 3 Department of Orthopaedic Surgery, Asan Medical Center, University of Ulsan College of Medicine, Seoul, South Korea

**Keywords:** Endocrinology, Molecular medicine, Oncology

## Abstract

Tumor-induced osteomalacia (TIO) is an uncommon paraneoplastic syndrome presenting with sustained hypophosphatemia. Treatment of choice is removal of the tumor causing the TIO, but identification of the culprit tumor by routine imaging is challenging. This study aimed to assess the usefulness of somatostatin receptor imaging, called ^68^Ga-DOTATOC PET/CT, in the management of patients with TIO. Twelve patients who were suspected of having TIO underwent ^68^Ga-DOTATOC PET/CT. Lesion detectability and maximum standardized uptake value (SUV_max_) were determined and retrospectively compared with the clinical/imaging surveillance and histopathologic diagnosis. The median duration of suspected TIO with hypophosphatemia was 7.8 years (range 2.1–21.0). Conventional radiologic and/or nuclear medicine images failed to identify the culprit tumors. However, ^68^Ga-DOTATOC PET/CT scans showed that 8 of the 12 patients had positive lesions, suggesting the presence of focal culprit tumors. The SUV_max_ of positive tumors was 1.9–45.7 (median: 11.5). Six skeletal lesions and two extra-skeletal lesions were identified. Seven of the lesions were pathologically confirmed as potential culprits of TIO. Hypophosphatemia was resolved in five patients who underwent lesion excision. The ^68^Ga-DOTATOC PET/CT is a useful whole-body imaging modality for the detection of causative tumors in patients with suspected TIO.

## Introduction

Tumor-induced osteomalacia (TIO), also known as oncogenic hypophosphatemic osteomalacia, is an uncommon paraneoplastic syndrome^1^. Up until now, less than 1000 cases of TIO were reported worldwide^2–7^. The overproduction of fibroblast growth factor-23 (FGF-23) by the culprit tumors is the primary causative mechanism in TIO^8^. The excess circulating FGF-23 causes chronic renal phosphate wasting and decreases the enzymatic activity of 1α-hydroxylase, which leads to sustained hypophosphatemia. The majority of culprit tumors accounting for osteomalacia originate from mesenchymal tumors; therefore, their usual location is bone or soft tissue layers^9^. Benign phosphaturic mesenchymal tumors and hemangiopericytoma are the most common tumor in TIO, whereas malignant osteosarcoma or fibrosarcoma are very rare.

Patients with TIO have ambiguous musculoskeletal symptoms, such as long-standing weakness, myalgia, fatigue, bone pain, and even fractures. Without a physician’s astute suspicion, a long delay between the first presentation and final diagnosis of TIO is frequent due to the non-specific symptomatology and scarcity of the disease. Although TIO is a very rare disease, the suspicion of TIO and detection of the culprit tumor is essential, given the long-term debilitating morbidity associated with aberrant bone metabolism. Moreover, the treatment of choice is the complete removal or ablation of the tumor^10,11^. Otherwise, permanent administration of phosphate supplement would be required for patients with TIO. However, the causative tumors are difficult to localize because tumors are small and slow-growing. Upon physical examination, most culprit tumors are rarely visible or palpable. Pinpointing the exact location of the culprit tumor is also very challenging using conventional radiologic and nuclear medicine images, including magnetic resonance imaging (MRI) and ^18^Fluoride-Fluorodeoxyglucose (^18^F-FDG) positron emission tomography/computed tomography (PET/CT)^12–16^.

The discovery of somatostatin receptor (SSTR) overexpression in mesenchymal tumors^17^, especially type 2 SSTR, prompted specific imaging of TIO patients using ^111^Indium-based octreotide scans, which are classic SSTR targeting scintigraphy^18^. State-of-the-art PET radiotracers, such as ^68^Ga-labeled DOTA (^68^Ga-DOTA)-based oligopeptides (DOTA^0^-Tyr^3^-Octreotate, ^68^Ga-DOTATATE; DOTA^1^-Nal^3^-octreotide, ^68^Ga-DOTANOC, and DOTA^0^-Phe^1^-Tyr^3^-octreotide ^68^Ga-DOTATOC) target the SSTR in neuroendocrine tumors^19^. Several recent reports demonstrated improved localization of culprit tumors in TIO using these PET radiotracers^13,20,21^. Although minor changes in amino acid sequences result in a differential affinity toward the type 2 SSTR families, the three tracers have similar performances in clinical practice^22^. Several reports have demonstrated the performance of ^68^Ga-DOTATOC PET/CT in these cohorts. In this context, we investigated the usefulness of ^68^Ga-DOTATOC PET/CT for the management of patients with TIO from a single Korean tertiary hospital.

## Materials and methods

### Study design and patient selection

The Institutional Review Board (IRB) in the Asan Medical Center (IRB no. 2019-1290) approved this study protocol and waived informed consent from patients due to the retrospective nature of the study. The study was conducted in compliance with the Declaration of Helsinki regulation. From June 2017 to May 2019, we retrospectively recruited 12 patients with suspicion of TIO who underwent ^68^Ga-DOTATOC PET/CT at the Asan Medical Center. Patient medical records were reviewed and documents regarding the commencement of clinical manifestations associated with TIO were retrieved. Additionally, the sequential biochemical laboratory results associated with bone metabolism and all the imaging studies, including radiology and nuclear medicine, were reviewed. When possible, we calculated the tubular reabsorption of phosphate (TRP) twice, according to the formula provided below, to evaluate the status of renal phosphate wasting^23^.

$$\text{TRP }=\left\{1- \frac{(serum \,creatinine \times urine \,phosphate)}{(urine\, creatinie \times serum \,phosphate)}\right\} \times 100.$$

### ^68^Ga-DOTATOC PET/CT imaging

The ^68^Ga-DOTATOC was manually synthesized with a ^68^Ge/^68^Ga generator (iThemba LABS, South Africa) and GMP grade DOTATOC (ABX, Germany) in the cyclotron laboratory of our institution^24,25^. Quality control was performed according to European Pharmacopeia; the synthesized ^68^Ga-DOTATOC satisfied all quality control criteria and the radiochemical purity of the ^68^Ga-DOTATOC was 98.2 ± 1.0%, as measured by HPLC. We injected a median dose of 4.65 mCi (range 3.4–5.4) ^68^Ga-DOTATOC, intravenously, in non-fasting patients. Sixty minutes after injection, PET/CT images were generated using the GE Discovery 690, 710, or 690 Elite systems. We acquired the emission PET scans at 3.5 min per bed. Nine patients underwent whole-body PET/CT from skull vertex to both feet. Three patients had torso PET/CT scan from the skull vertex to the proximal thigh. The lesion detectability and SUV_max_, normalized to body weight, were determined. Additionally, we compared the ^68^Ga-DOTATOC PET/CT results with the outcomes of clinical/imaging surveillance and/or pathology.

## Results

Table 1 summarizes the patient characteristics. We recruited 12 patients (four males and eight females) with a median age of 60 years (range 47–78). During the initial visit to our hospital, all patients had overt hypophosphatemia (serum phosphate ranging from 0.9 mg/dL to 2.4 mg/dL). Nine of ten patients had TRP levels below 85%, suggesting hypophosphatemia of renal origin. We measured FGF-23 in only two patients; both patients had abnormally increased FGF-23 values that were specifically suggestive of TIO.

**Table 1:** Patient characteristics.

	Sex	Age	Duration (years)	P (2.5–4.5 mg/dL)	Ca (8.6–10.2 mg/dL)	ALP (40–120 IU/L)	Cr (0.7–1.4 mg/dL)	PTH (10–65 pg/mL)	FGF23 (> 180 RU/mL)	TRP (> 85%)	25 (OH)D (8.0–51.9 ng/mL)	1.25 (OH)_2_D (19.6–54.3 pg/mL)	MRI/CT	^18^F-FDG PET	^68^Ga-DOTATOC PET detection (FOV)	Lesion (SUV)	Longest diameter (cm)	Pathology	Surgery	Name of surgery
1	F	74	21.0	1.6	8.9	667	0.50	55.6	x	78/83	10.8	21.8	x	x	Positive (W)	Maxilla (12.7)	1.8	Positive	O	Partial maxillectomy
2	M	54	2.0	2.1	8.6	183	0.95	51.3	x	78/82	x	x	O	O	Positive (W)	Groin (24.0)	1.1	Positive	O	Lymph node excision
3	F	71	12.1	1.4	8.9	407	0.51	127.0	x	57	48.9	x	O	x	Positive (W)	Thigh (9.9)	6.5	Positive	O	Wide excision
4	F	47	11.2	1.0	8.5	125	0.50	71.6	x	84/80	56.6	x	O	x	Positive (W)	Fibula (1.9)	1.6	Positive	O	Resection of fibular head
5	M	53	2.1	2.4	8.8	91	0.87	41.7	x	83/82	19.6	x	O	x	Positive (T)	L3 (4.5)	1.4	Positive	O	Partial corpectomy and bone graft
6	F	60	2.3	0.9	8.7	197	0.44	26.6	412	x	23.0	12.5	O	O	Positive (W)	C2 (10.7)	2.8	Positive	O	Curettage and bone graft
7	F	48	18.8	1.5	8.3	361	0.90	56.7	3740	67	2.4	x	O	x	Positive (W)	Femur (45.7)	4.2	Positive	x	x
8	M	70	7.3	2.1	9.2	205	0.80	26.6	x	70	21.0	7.0	O	O	Positive (W)	Pubic (12.2)	2.1	x	x	x
9	F	63	8.3	2.1	9.0	168	1.1	29.8	x	x	35.6	39.7	O	x	Negative (W)	x	x	x	x	x
10	F	60	8.3	1.8	7.0	84	0.40	153.0	x	90/87	1.5	x	O	x	Negative (T)	x	x	x	x	x
11	F	48	6.5	1.1	8.8	169	0.83	78.1	x	74/72	3.5	11.1	O	O	Negative (W)	x	x	x	x	x
12	M	78	3.9	1.6	8.5	283	0.64	54.8	x	82/79	28.7	14.1	O	O	Negative (T)	x	x	x	x	x

*P* phosphorous, *Ca* calcium, *ALP* alkaline phosphatase, *Cr* creatinine, *PTH* parathyroid hormone, *FGF-23* fibroblast growth factor-23, *TRP* tubular reabsorption of phosphate, *25(OH)D* 25-hydroxyvitamin D,*1,25(OH)*_*2*_*D*,1,25-dihydroxyvitamin D, *MRI* magnetic resonance imaging, *CT* computed tomography, ^*18*^*F-FDG* ^18^Fluoride-Fluorodeoxyglucose, *PET* positron emission tomography, ^*68Ga*^*-DOTATOC PET/CT* ^68Ga^-labeled DOTA^0^-Tyr^3^ octreotide (DOTATOC), *FOV* field of view, *SUV* standardized uptake value, *F* female, *M* male, *W* whole body, *T* Torso, *C2* 2nd cervical spine, *L3* 3rd lumbar spine.

**Figure 1:**
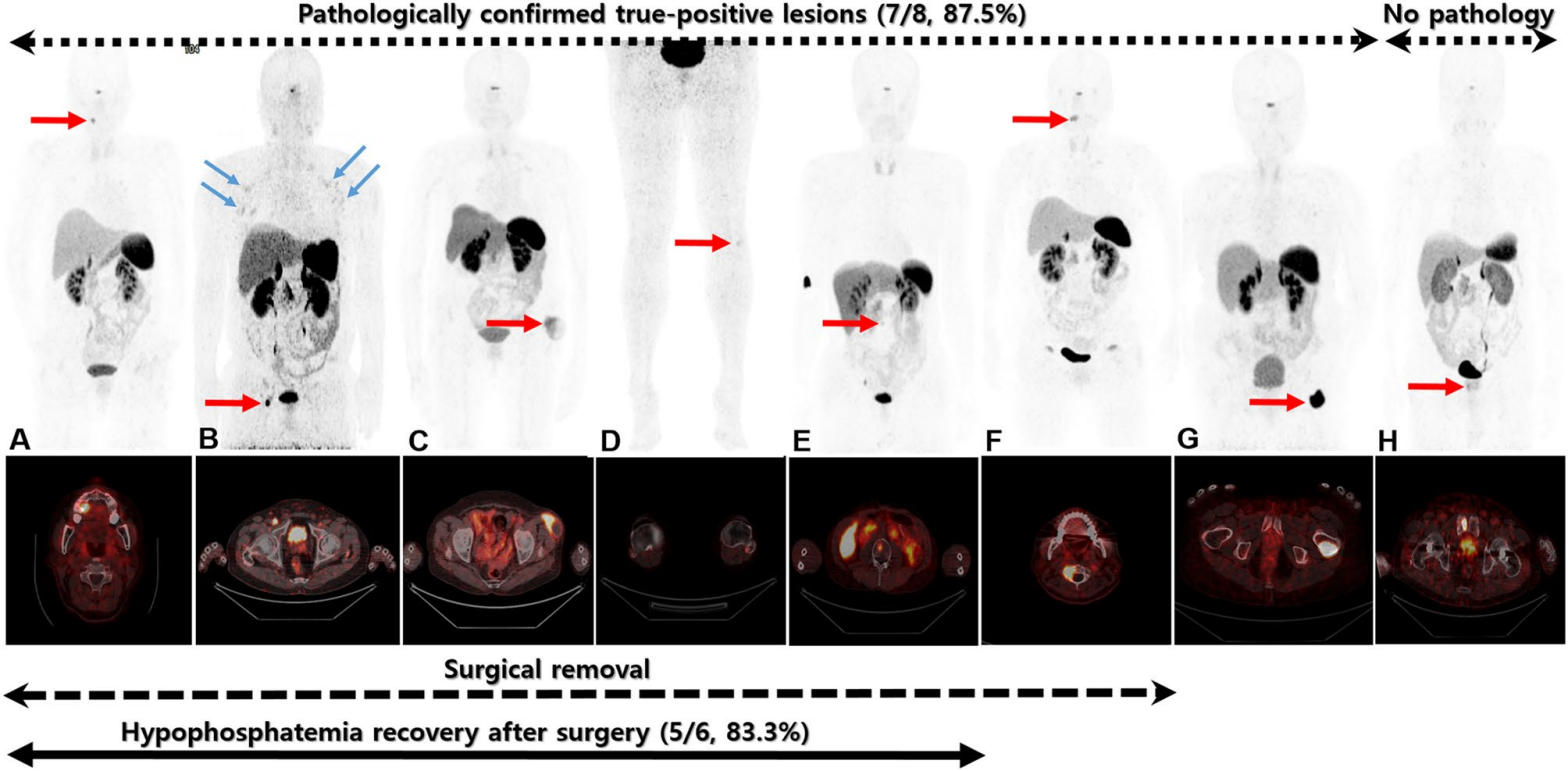
Maximum intensity projection (MIP) images and axial PET/CT images of eight patients with positive lesions according to the ^68^Ga-DOTATOC PET/CT. The red arrows in the MIP images indicate causative lesions in the corresponding PET/CT image, whereas the blue arrows in (**B**) indicate non-pathognomonic uptake. (**A**) Lesion in the right maxilla with SUV_max_: 12.7. (**B**) Lesion in the right inguinal lymph node with SUV_max_: 24.0. (**C**) Lesion in the left thigh with SUV_max_: 9.9. (**D**) Lesion in the left fibula with SUV_max_: 1.9. (**E**) Lesion in the L3 body with SUV_max_: 4.5. (**F**) Lesion in the C2 body with SUV_max_: 10.7. (**G**) Lesion in the left femur with SUV_max_: 45.7. (**H**) Lesion in the right pubic bone with SUV_max_: 12.2.

**Figure 2:**
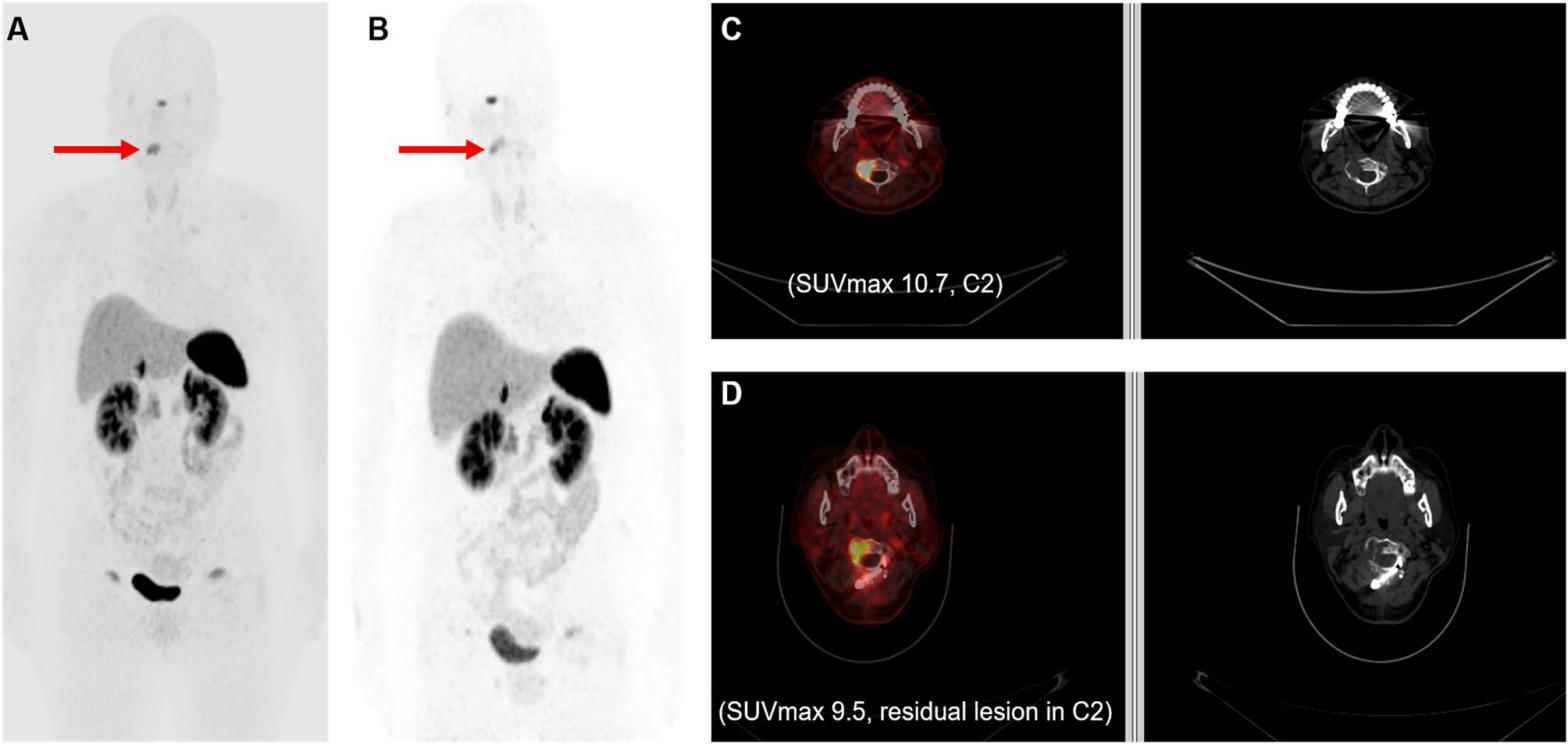
A case of unresolved hypophosphatemia after surgical excision was demonstrated by the follow-up with ^68^Ga-DOTATOC PET/CT. (**A**) MIP image of initial ^68^Ga-DOTATOC PET/CT with the red arrow indicating the culprit lesion; (**B**) MIP image of follow-up with ^68^Ga-DOTATOC PET/CT 6 months after surgery with the red arrow indicating the residual culprit lesion; (**C**) axial PET/CT and non-enhanced CT images of culprit lesion in initial ^68^Ga-DOTATOC PET/CT; (**D**) axial PET/CT image and non-enhanced CT images of residual culprit lesion in follow-up ^68^Ga-DOTATOC PET/CT.

The median duration of suspected TIO was 7.8 years (range 2.1–21.0 years), reflecting the long-standing disease courses in most patients. During the period of suspected TIO, conventional radiologic (X-ray, CT, and MRI) and/or nuclear medicine images (bone scan and ^18^F-FDG PET/CT) failed to pinpoint the culprit lesions. Even though some patients demonstrated several tumors in bones or soft tissues, those tumors were considered non-specific benign primary tumors by the interpreting radiologist or nuclear medicine physician. However, 8 of the 12 patients (66.6%) had focal positive uptake of ^68^Ga-DOTATOC in areas other than the physiologic uptake in the pituitary gland, adrenal glands, and uncinate process of the pancreas, thereby suggesting the possibility of culprit tumors (Fig. 1). Regarding the quantitative parameters of the eight positive tumors, SUV_max_ ranged from 1.9 to 45.7 (median: 11.5) and the longest tumor diameter was measured between 1.1 and 6.5 cm (median 1.95 cm). Six skeletal lesions were located in the second cervical vertebral body (C2), third lumbar vertebral body, pubic bone, femur, maxilla, and fibula and two extra-skeletal lesions were located in the groin and thigh. Among the eight patients with positive tumors, seven patients (87.5%) were pathologically confirmed and six patients finally underwent surgery to remove the tumor pinpointed by the ^68^Ga-DOTATOC PET/CT. Phosphate levels completely recovered in five out of six patients after the surgery without requiring phosphate supplementation. One patient with a C2 vertebral mass underwent a second ^68^Ga-DOTATOC PET/CT 6 months after the operation because of unresolved hypophosphatemia (Fig. 2). Compared to the basal ^68^Ga-DOTATOC PET/CT, the second scan showed slightly decreased but residual ^68^Ga-DOTATOC uptake by a persistent osteolytic lesion in the operative bed. Thus, the reason for the unresolved hypophosphatemia was a remnant tumor.

**Figure 3:**
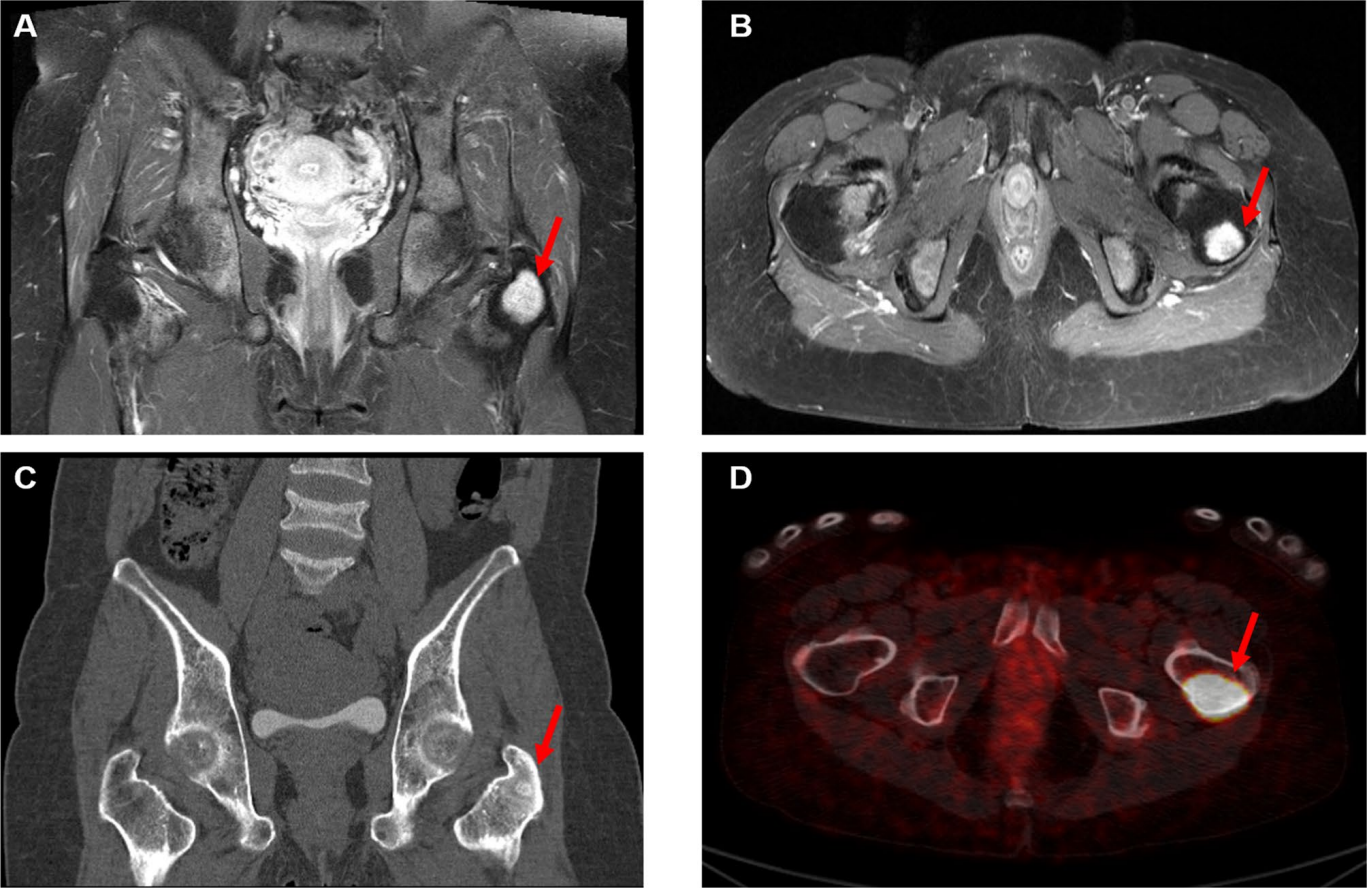
A representative case showing the specificity and strength of ^68^Ga-DOTATOC PET/CT as compared to conventional images. A 48-year-old woman suspected of TIO had pelvic CT and MRI 10 years before undergoing the ^68^Ga-DOTATOC PET/CT because of hip pain. The images showed a well-enhancing mass-like sclerotic lesion in the left femur, which was in the greater trochanter in the coronal, axial enhanced pelvic MRI (**A**,**B**) and coronal non-enhanced pelvic CT images (**C**). However, the lesion was considered a hematopoietic marrow or Brown tumor at that time rather than a primary bone tumor. Ten years later with ^68^Ga-DOTATOC PET/CT image (**D**), the lesion showed slowly grown tumorous features with intense radiotracer uptake strongly suggesting the culprit tumor for osteomalacia. Subsequent bone biopsy favored the presence of a phosphaturic mesenchymal tumor.

Two patients, who had focal positive uptake in ^68^Ga-DOTATOC PET/CT, did not undergo surgery. A 48-year-old female patient (Number 7, Fig. 3), with bone biopsy results showing a phosphaturic mesenchymal tumor on her femur, refused curative surgery because of good compliance to phosphate supplement, albeit proportionally increased phosphate demand as the mass grows. A 70-year-old male patient (Number 8) with a focal pubic bone lesion also refused to undergo biopsy or surgery, despite sustained medication. Since the patient had previous surgeries of both femurs due to prior fragility fractures, the patient was concerned about post-operative complications.

Among the four patients with negative ^68^Ga-DOTATOC PET/CT results, two patients seemed to have plausible clinical conditions, which excluded the possibility of TIO. One patient had a long history of the anti-viral medication, adefovir, due to chronic hepatitis B virus infection. This patient history implied an “adefovir-induced hypophosphatemic osteomalacia.” Another patient had a history of celiac disease in which intestinal phosphate absorption is reduced. Concordantly, the patient’s calculated TRP value was above 85%. The remaining two patients with negative ^68^Ga-DOTATOC PET/CT results did not have clear reasons for persistent hypophosphatemia, and they were given phosphate supplements.

## Discussion

In this study, we detected the culprit tumors that induced osteomalacia in seven out of nine patients whose calculated TRP was suggestive of renal hypophosphatemia^26^. Five out of six patients underwent excision of the tumor identified by ^68^Ga-DOTATOC PET/CT and serum phosphate levels returned to normal after the surgery. In two patients, the positive lesions identified by PET/CT had elevated FGF-23 levels. Another two patients with less probability of TIO showed no evident lesions using PET/CT, and no false positive case was observed during comparisons with the biopsy results.

Although alternative radiotracers are being used for SSTR PET imaging, several investigations reported successful results in more than a dozen patients with TIO. Utilizing the same radiotracer as our study, Paquet et al. showed a 60% detection rate in 15 patients who had suspected TIO, affecting the patient management in 67% of the cases^27^. Of note, one true positive patient without elevated FGF-23 levels was identified in the Paquet study. With ^68^Ga-DOTATATE, Zhang et al. showed 100% sensitivity, 90.9% specificity, and 97.7% accuracy for detecting the culprit lesions in 54 patients^28^. Only one false positive case was detected and the ten patients with negative ^68^Ga-DOTATATE PET/CT were all true-negative at follow-up. However, Zhang et al. did not offer any supportive laboratory data about the level of TRP or FGF-23. Singh et al. demonstrated promising results in 17 patients using ^68^Ga-DOTANOC^29^. Among the nine patients who had suspicions for culprit lesion according to the PET/CT, seven were confirmed to have those lesions. In addition, the Singh study focused on unraveling the other uptake induced by fracture or degenerative changes beyond the strength of detecting the culprit lesion. We also observed such uptake, usually induced by fracture (Blue arrows in Fig. 2B highlight the rib fractures). Those lesions exhibited a mild uptake with symmetric distributions, which contrasted with the unilateral manifestation of most of the culprit lesions. Therefore, close examination is recommended for interpretation of the ^68^Ga-DOTA-based PET radiotracers in patients who have concomitant fractures.

Sincere efforts have been made to reveal the culprit tumors in patients with TIO using various imaging modalities other than specific SSTR scan, including simple radiography, bone scan, CT, MRI, and ^18^F-FDG PET/CT^14–16,30^. However, almost all conventional modalities showed low performance due to the non-pathognomonic findings or small tumor size. This could be the reason for inappropriately delaying the diagnosis of TIO. Indeed, we also had patients whose culprit tumors were observed on CT, MRI, or ^18^F-FDG PET/CT a few years before the ^68^Ga-DOTATOC PET/CT. However, the presence of culprit tumors was not specifically demonstrated at that time and the opportunity for early detection was lost. Figure 3 presents one such representative case.

In this context, the specificity for SSTR with “one-shot” whole-body functional imaging available by ^111^Indium octreotide scintigraphy is considered as a mainstay in assessing patients with TIO^18,31^. However, superior spatial resolution inherent in the PET/CT system, better pharmacokinetics, and a higher affinity toward SSTR by shorter half-life PET radiotracers with a cyclic chelator render ^68^Ga-DOTA-based PET radiotracers the imaging of choice not only for patients with neuroendocrine tumors but also for patients with TIO^13,17^.

The evaluation of treatment response or detection of the recurred/remnant tumor using post-treatment ^18^F-FDG PET/CT is a popular approach in the field of oncology^32^. To the best of our knowledge, we have shown, for the first time, the additional value of follow-up with ^68^Ga-DOTATOC PET/CT after the basal scan to detect residual tumors whose hypophosphatemia persists even after surgery. Our results provide another option in the management of patients with TIO using ^68^Ga-DOTA-based PET/CT beyond the pivotal role of detecting the primary occult tumor. Previously, a case that also showed the usefulness of detecting the residual culprit tumor surgery using ^68^Ga-DOTANOC PET/CT was reported, but an initial PET/CT for comparison was not available^33^.

There are a few limitations to this study. First, even though FGF-23 is a key hormone and a sensitive indicator of TIO, testing for FGF-23 is not available in clinical practice in our country, preventing us from getting FGF-23 results. For this reason, we had just two patients who had the serum FGF-23 test. Easy accessibility to FGF-23 assays would be helpful for the early detection of TIO. Nevertheless, the results of ^68^Ga-DOTATOC PET/CT in this study were not affected by the results of the FGF-23 assay. Second, not all patients underwent whole-body PET/CT imaging, including that of the lower extremity, due to our inexperience. Given the positive lesion detected in the fibula of one patient, whole-body PET/CT imaging rather than torso imaging is recommended in these patients. Third, in the patients who had negative ^68^Ga-DOTATOC PET/CT without definite plausible reasons, we did not perform any gene mutation tests. Hypophosphatemia-related genetic diseases, such as X-linked hypophosphatemic rickets or autosomal dominant hereditary hypophosphatemic rickets^34–36^, are very rare and but cannot be excluded. Finally, the total number of patients is quite low because of the rarity of the disease and limited accessibility of ^68^Ga-labeled radiopharmaceuticals. Due to the small number of patients included in this study, we omitted the statistical analysis regarding the diagnostic performance of ^68^Ga-DOTATOC PET/CT.

In summary, ^68^Ga-DOTATOC PET/CT appears to be a very robust and innovative imaging modality for detecting causative tumors in patients with suspected TIO and for detecting remnant or recurred tumors after surgery.
